# An Improved Entropy-Weighted Topsis Method for Decision-Level Fusion Evaluation System of Multi-Source Data

**DOI:** 10.3390/s22176391

**Published:** 2022-08-25

**Authors:** Lilan Liu, Xiang Wan, Jiaying Li, Wenxi Wang, Zenggui Gao

**Affiliations:** 1 School of Mechatronic Engineering and Automation, Shanghai University, Shanghai 200444, China; 2 Shanghai Key Laboratory of Intelligent Manufacturing and Robotics, Shanghai University, Shanghai 200444, China; 3 Aerospace System Engineering Shanghai, Shanghai 201108, China

**Keywords:** multi-source data, decision-level fusion, fusion evaluation system

## Abstract

Due to the rapid development of industrial internet technology, the traditional manufacturing industry is in urgent need of digital transformation, and one of the key technologies to achieve this is multi-source data fusion. For this problem, this paper proposes an improved entropy-weighted topsis method for a multi-source data fusion evaluation system. It adds a fusion evaluation system based on the decision-level fusion algorithm and proposes a dynamic fusion strategy. The fusion evaluation system effectively solves the problem of data scale inconsistency among multi-source data, which leads to difficulties in fusing models and low fusion accuracy, and obtains optimal fusion results. The paper then verifies the effectiveness of the fusion evaluation system through experiments on the multilayer feature fusion of single-source data and the decision-level fusion of multi-source data, respectively. The results of this paper can be used in intelligent production and assembly plants in the discrete industry and provide the corresponding management and decision support with a certain practical value.

## 1. Introduction

### 1.1. Development and Levels of Data Fusion

#### 1.1.1. The Development and Significance of Data Fusion

The process of human cognition of the environment begins with the acquisition of multiple forms of redundant and complementary information by means of perception, such as sight, sound, touch and taste. This information is then processed by the brain to obtain human understanding and perception of things. This process of moving from perception to cognition is the process of fusing information from multi-sources [1]. Since early research in information fusion was aimed at the field of data processing, it was also known as multi-source data fusion, which is its most widely and commonly used name. In research, scholars also often use, for example, multi-sensor fusion [2], multi-modal fusion and other similar terms. These fusion concepts are common in research thinking and methodology, and current academic research does not make clear and strict distinctions. This paper also uses the name multi-source data fusion, which is a widely used and common name [3].

Data fusion research first originated in the military field, and foreign research on data fusion is more comprehensive and advanced. The first use of data fusion methods by Bar-Shalom in the 1970s led to a significant improvement in the performance of target detection and tracking [4]. In the 1980s, the Americas began to develop tactical surveillance systems for data fusion. Durrant-Whyte proposed the use of multi-sensor data fusion, which can effectively improve target recognition performance [5]. In the early 1990s, the JDL (Joint Directors of Laboratories) in the Americas gave a widely accepted definition of data fusion [6]. The current data fusion systems developed and matured in the America and Britain mainly include an all-source information analysis system, enemy situation analysis system, artillery intelligent data fusion system, and so on, which have high maturity and advanced performance. The study of data fusion in China started late and it was not until the outbreak of the Gulf War in the late 1980s that it attracted wide attention in China. The first data fusion conference was held in China in 1995, which was organized by the national science and industry commission. With funding from the government, the military and institutions, universities and research institutes in China have begun research, and a large number of research results have emerged [7,8]. Due to the large gap compared to the international advanced level, the development of new generation of domestic application systems and the rapid development of civil technology have posed more challenges to data fusion.

Since Germany put forward its Industry 4.0 strategy in 2014, countries such as the US, Japan and China have also launched their own national development implementation strategies for manufacturing. The core technology of the strategies of all countries can be summarized as the conversion of data into intelligence through data fusion technologies. Due to the rapid development of industrial internet technology, the traditional manufacturing model and industrial ecology has been greatly transformed, and digital transformation is urgently needed. Data are the core asset of an enterprise, and the flow of data is critical and decisive throughout all processes. Where digitalization solves the problem of data sources, networking solves the problem of data flow, and intelligence solves the problem of autonomous data flow. The series of processes that combine these aspects of the three rely on the extraction and fusion of the knowledge implicit in the multi-source data. This is not only a hot issue in computer science, but also a key to achieving the transformation of China’s manufacturing industry and the significance of this paper’s research [9].

#### 1.1.2. Levels of Data Fusion

Data fusion is the utilization of the complementary information between different data sources to obtain a more complete and adequate representation and to enhance the performance and explainability of the fused model [10,11]. Existing data fusion techniques can be divided into three kinds according to the level of fusion, namely, data-level fusion, feature-level fusion, and decision-level fusion [12].

Data-level fusion is the lowest level of fusion that means the fusion of the collected raw data after simple pre-processing, including weighted averaging, wavelet transform [13], HIS transform [14], and so on. This type of fusion method is simple and easy to process for large volume data. Its main advantages are low loss of data information and high fusion accuracy. However, the algorithm has obvious limitations due to the large amount of data processed, poor real time performance and the requirement to process data of the same type.

Feature-level fusion methods are the direct serial fusion of features from all data sources. Such methods include Kalman filtering, the entropy theory method [15], the artificial neural network method [16], deep learning [17] and other methods. It has the advantage of achieving data compression, which facilitates real-time data processing, but loses some of the lower-level data information and has reduced fusion performance [18]. As different data sources have different representations, distributions and densities, this leads to the problem of small sample data, which makes it difficult to construct fusion models, and the fusion results are dominated by the data source with more data [19].

Decision-level fusion originated in the field of pattern recognition and is also known as semantic-level fusion [20]. It uses different algorithms for different data sources to build recognition models. The recognition results are then combined in a decision-level to give a conclusion. The aim of decision-level fusion is to overcome the problems of redundancy, missing, interference and uncertainty in the raw data [21]. Traditional fusion methods include Bayesian inference [22], expert systems, D-S evidential reasoning [23], and fuzzy set theory [24,25]. In the field of deep learning, decision-level fusion methods have also been proved to be significantly more robust than feature-level fusion methods [26,27].

From the above study, it is clear that compared to feature-level fusion and decision-level fusion, data-level fusion is less stable, and the fused data requires the same type of data and the algorithm is less robust. Current research on multi-source data fusion therefore focuses on feature-level fusion and decision-level fusion [28].

### 1.2. Current Research Status and Challenges of Multi-Source Data Fusion

Most traditional multi-source fusion methods use simple voting, averaging and ranking methods, which are simple and easy to implement for modelling, but the performance of the fused model is poor. Therefore, the current mainstream multi-source fusion method uses ensemble learning as the guiding idea for fusion. Xiao designed a combination strategy of ensemble learning, which combines a KNN (K-nearest Neighbor) with an SVM (Support Vector Machine) to obtain a better fusion output and improve the robustness of the model [29]. Amin et al. used the TOPSIS method for weighted decision scoring of each feature [30]. Zhang et al. proposed a TOPSIS intuitionistic fuzzy decision making algorithm based on category similarity weight [31]. This type of fusion method does not consider the performance advantages and disadvantages of each independent model under different data sets, but simply fuses the results of the models directly. The final performance of the fusion model will then depend heavily on the quality of the original data before the performance of the independent models, which is clearly unscientific.

To address these problems, Shang et al. proposed a multi-scale deep feature fusion method based on information entropy [32]. Methods based on similar principles, such as uncertainty weighting [33], dynamic weight averaging [34], and dynamic evidential fusion [35]. These improved methods, while giving different weights to each model according to its performance, solve the problem of inconsistent data scales, making it difficult to fuse the models. However, due to the presence of poorly performing models, the fused model has more irrelevant information, insufficient representation capability and low accuracy after fusion. Even the performance is significantly lower compared to the performance of the single model before fusion [36]. Through the analysis of the current situation of domestic and foreign research and the hierarchy of data fusion, we found that scholars have made remarkable progress in multi-source data fusion, but the current multi-source data fusion methods mainly focus on the field of algorithm optimization. There are two main problems with these methods.

1. The existing algorithms do not take into account the characteristics of the data and the long-tail distribution of the data and the uneven difficulty of sample identification due to small sample data. The direct fusion of data from multi-sources leads to poor model performance.
2. Inconsistent data scales between multi-source data make it difficult to fuse the models and the low accuracy of the fused model.

Through the study of these two aspects, this paper addresses the difficulties of multi-source data fusion in industrial production applications, realizes digital management and intelligent decision-making of multi-source data fusion, and supports the digital transformation of China’s manufacturing industry.

### 1.3. Scope of Our Work and Contribution

Synthesizing the previous studies, this paper proposes an improved entropy-weighted topsis method of multi-source data fusion evaluation system, whose main contributions are summarized as follows.

1. For the characteristics of local correlation of image data and sensing data, this paper designs the maximum average pooling (Max-AVG pooling) feature extraction layer and residual dilated convolution feature extraction layer, respectively. This paper combines the proposed transfer learning VGG19 multilayer feature fusion network to solve the problem of small sample data, to achieve the multilayer feature fusion of data and to improve the robustness and accuracy of the model.
2. In response to the problem of inconsistent data scales of multi-source data, which makes it difficult to fuse models and poor performance after fusion, research on decision-level fusion methods for multi-source data has been carried out. In this paper, an improved entropy-weighted topsis method fusion evaluation system and dynamic fusion strategy are proposed to eliminate the models with poor performance before fusion.

**Figure 1:**
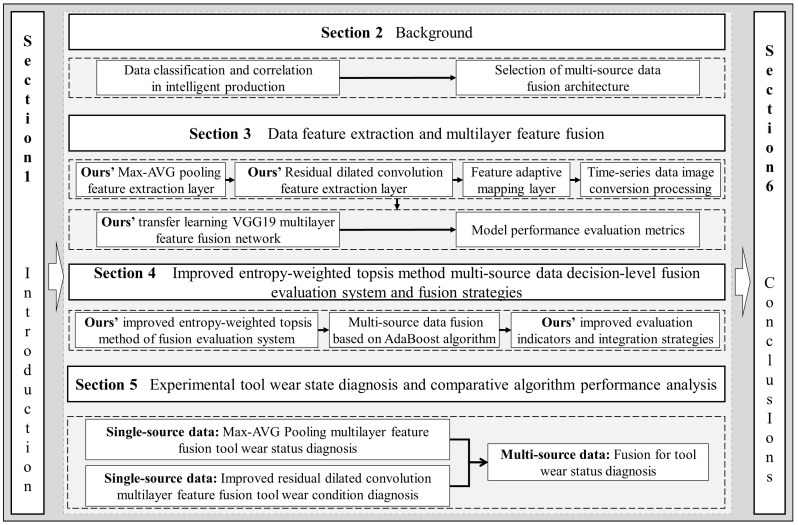
Overview of the structural framework and the research process of this paper.

The remainder of this paper is organized as follows. In Section 2, we analyze the categories and associations of data in intelligent production and introduce the multi-source data fusion architecture and fusion methods. In Section 3, feature extraction and multilayer feature fusion on a single data source are presented. Our findings are the design of two feature extraction layers and the proposal of a transfer learning VGG19 multilayer feature fusion network. In Section 4, we propose an improved entropy-weighted topsis method fusion evaluation system, improved evaluation metrics and fusion strategies, and undertake a study on decision-level fusion of multi-source data. In Section 5, the experiments in this section are divided into the experimental validation of multilayer feature fusion on a single data source and decision-level fusion on multi-source data. Experimental verification is undertaken, respectively. The algorithm and the fusion evaluation system proposed in this paper are compared with the current mainstream fusion methods using tool wear experiments as an example, and the effectiveness of the proposed method is verified. Section 6 concludes this paper with some future research directions. In Figure 1, we show the structural framework and the research process of this paper.

## 2. Background

### 2.1. Data Classification and Correlation in Intelligent Production

#### 2.1.1. Data Sources and Modality Classification

Data in intelligent production is an overall reference to the various types of multi-source data generated in industry around the full product lifecycle of intelligent manufacturing process. Multi-source data are data from different sources that are collected in one dataset [37]. The object of this paper is the multi-source data generated in the smart shop. Namely, products and devices are in the internet of things, and all kinds of data are generated in real time. This type of data, which reflect the operational status of equipment and products, is also called industrial big data in a narrow scope [38]. Multi-modal data are not limited to modal data such as text, images, audio and video, but also include mixed digital data provided by various sensors, systems and devices [39]. Therefore multi-modal data can be summarized as data of the same origin and of interrelated but different types.

#### 2.1.2. Sources of Data and Correlation between Modalities

**Figure 2:**
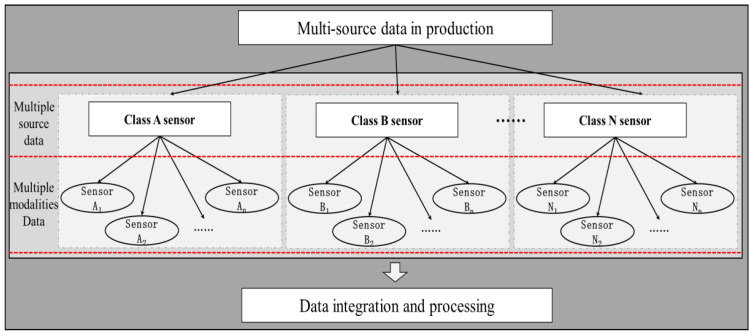
Correlating with multi-source data and multi-modal data.

The data sources and modal classification criteria for intelligent production show that there is a correlation between multi-source and multi-modal [40]. Based on the previous studies, it is clear that multi-source data are data collected by different types of sensors, while multi-modal data are data collected by several different sensors of the same type [41,42]. According to the combination of multi-source data and the relationships between multi-modal data shown in Figure 2, multi-source data can be classified into four types: single-source single-modal data, single-source multi-modal data, multi-source single-modal data, and multi-source multi-modal data.

Multi-source multi-modal data are all types of production data collected by several different sensors of different types, and are also referred to as multi-source data. In practice, the data may be unavailable due to noise and large-scale missing issues that cannot be removed from different modalities and sources. As multi-source data can provide more information than single-source data, by complementing and correcting each other’s data, they can provide more accurate information to validate the above problems. Therefore, the research on the fusion of multi-source data is also a hot and difficult topic in the current academic research.

### 2.2. Selection of Multi-Source Data Fusion Architecture

#### 2.2.1. Multi-Source Data Fusion Architecture

Multi-source data fusion focuses on how to fuse multiple data with certain architectures and methods to achieve a unified representational framework that contributes jointly to the target task. In this paper, the fusion architectures for multiple sources are directly divided into three types: joint architecture, collaborative architecture, and encoder and decoder architecture [43].

(1) Joint structure.

The strategy of the joint architecture is to map the coded representations of information from each single data source into the same shared semantic subspace in order to fuse different types of features. The core idea is the association of features through feature weighting or certain rules to achieve the union of features. The joint architecture has the advantage over other architectures of having a simple integration method and a shared space with semantic invariance. However, this architecture requires a high level of semantic integrity of information across data sources, and problems of incomplete or inconsistent data can be magnified in the later stages of fusion, affecting the effectiveness of the fusion.

(2) Collaborative architecture.

The collaborative architecture is the synergy of information from a single data source with each other through the action of different constraints. The goal of the collaborative architecture is to transfer knowledge and coordinate associations across data source domains. Because of the differences between the information from the data sources, the collaborative architecture can maintain characteristics unique to each data. Collaborative architectures have the advantages of independent operation of models constructed on each data source and low quality requirements for data sources, and are widely used in cross-modal learning [44]. However, such architectures are difficult to fuse, and implementing transfer learning across multiple modes requires specifically designed fusion methods.

(3) Encoder and decoder architecture.

The encoder and decoder architecture is used when mapping one data source modality to another target space modality transformation and consists of two parts, the encoder and the decoder. This architecture has the advantage of being able to generate new target space samples based on the source modalities, however each encoder and decoder can only encode one type of data. This architecture can be seen as a special collaborative architecture designed for specialized data, with a complex and poorly generalized module design.

#### 2.2.2. The Selection of Fusion Architectures and Methods

(1) Selection of fusion architecture.

From the above study of the three fusion architectures, it is clear that each has its strengths and weaknesses and that there is no one architecture that is completely universal and performs well for all data. One of the problems facing multi-source data fusion is how to choose the appropriate architecture based on the characteristics of the multiple sources of data in production. A comparison of the advantages and disadvantages of different fusion architectures is shown in Table 1.

Due to the special character of industrial big data, which is highly susceptible to noise, missing data and data anomalies, it is not suitable for fusion using a joint architecture model built on a unified structure. There are primary and secondary relationships between multi-source data collected at different ports. Establishing models that run independently on each data source not only maintains the unique characteristics between the data, but also allows the selection of appropriate algorithms to model the data in accordance with the characteristics of that data source [45,46]. The fusion in intelligent production emphasizes the collaborative sharing of multiple information, requiring low difficulty in constructing the architecture, low difficulty in fusion and good data quality. So the paper chooses a collaborative architecture for the fusion of multiple sources of data in production.

(2) Selection of fusion method.

Feature-level fusion methods take full account of feature interactions and correlations between data sources and have less information loss and model performance loss, but require longer data processing times and are more sensitive to data quality problems. Decision-level fusion methods are well adapted to the problems of low data quality and missing data in industrial data, although the loss of information and model performance is high. Therefore, the fusion method in this paper uses decision-level fusion methods.

**Figure 3:**
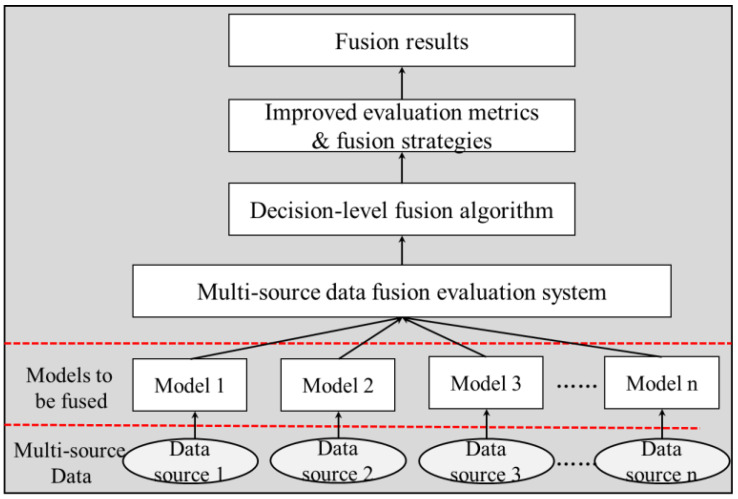
The fusion process of a multi-source data fusion evaluation system.

Deep learning has also played a powerful role in multi-source data fusion methods in recent years, and some of the literature also refers to deep learning multi-source data fusion models as model-based fusion methods [43]. These fusion models are able to handle multiple tasks simultaneously on a unified model structure with clear roles for each module. However, data features from different sources do not distinguish between importance and weights, which makes the mere use of multi-source data for fusion not necessarily improve model performance [47,48,49]. Therefore, the multi-source data fusion method in this paper does not use model-based fusion methods represented by deep learning. Based on the above problems, this paper proposes to add a fusion evaluation system to the original fusion algorithm. The fusion is carried out for the performance differences of each independent model on different data sources, and when there are data sources that significantly impair the performance of the fusion model they are removed from the fusion system. The fusion process of the multi-source data fusion evaluation system is shown in Figure 3.

## 3. Data Feature Extraction and Multilayer Feature Fusion

### 3.1. Data Feature Extraction and Data Transformation for Deep Learning

With the rise of artificial intelligence, deep learning feature extraction algorithms are gradually replacing traditional feature extraction algorithms. Deep neural networks pair convolutional kernels of different sizes with activation functions in order and hierarchy to build different feature extraction and activation layers to complete automatic feature extraction. The feature extraction algorithms of deep learning are highly adaptable and accurate, and the learned features have certain semantic characteristics, which makes deep learning successful not only in the field of computer vision, but also in fault diagnosis [50], acoustic event classification and wind power prediction [51]. In this section, based on the local correlation characteristics of image data and sensing data, the residual connection layer [52] and the dilated convolution layer [53] are introduced. The maximum average pooling (Max-AVG pooling) feature extraction layer for multiple channels and the dilated convolution feature extraction layer for residual connection are designed according to the characteristics of local correlation of image data and sensor data, respectively.

(1) Multi-channel Max-AVG pooling feature extraction layer.

**Figure 4:**
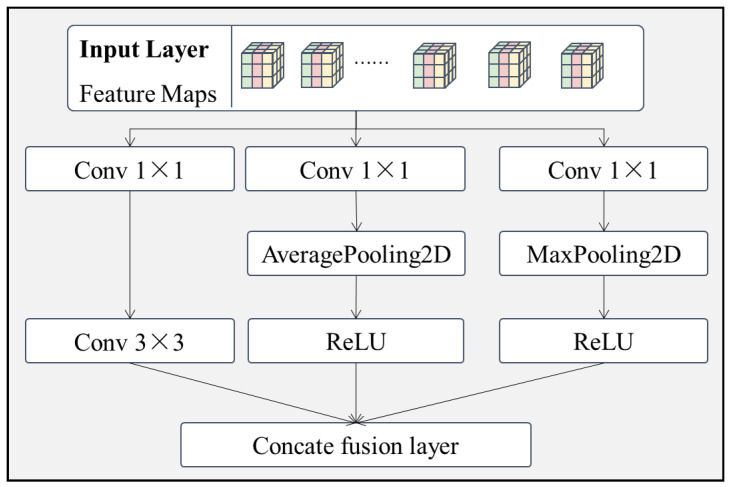
Structure of the Max-AVG pooling feature extraction layer.

By observing the image of the surface of the target to be inspected, it can be seen that the surface defect areas are brighter or darker than the defect-free areas. Based on the above problem, a maximum average feature extraction module is designed in this subsection. The module has three branches, as shown in Figure 4.

- Branch 1.
  The original features are passed sequentially through a convolutional layer of size 1×1 and a convolutional layer of size 3×3. The branch has not been specially processed, so branch 1 enriches the original features by increasing the number of channels in the convolutional kernel while keeping the original image features as much as possible.
- Branch 2.
  The original features are passed through a convolutional layer of size 1×1 and an average pooling layer of size 2×2 in turn, and finally regularized by connecting the ReLU activation layer. Branch 2 uses the averaging pooling layer mainly to filter out noisy data from the original features and to enhance the robustness of the model.
- Branch 3.
  The original features are sequentially passed through a convolutional layer of size 1×1 and a maximum pooling layer of size 2×2, and finally connected to a ReLU activation layer for regularization. Branch 3 uses the maximum pooling layer, mainly to extract the foreground features with higher brightness from the original features, so as to better distinguish the background from the foreground in the image.

The raw feature data are processed in 3 different branches and finally connected by the concate fusion layer by channel to provide the fused feature data for the later model.

(2) Residual dilated convolution feature extraction layer.

**Figure 5:**
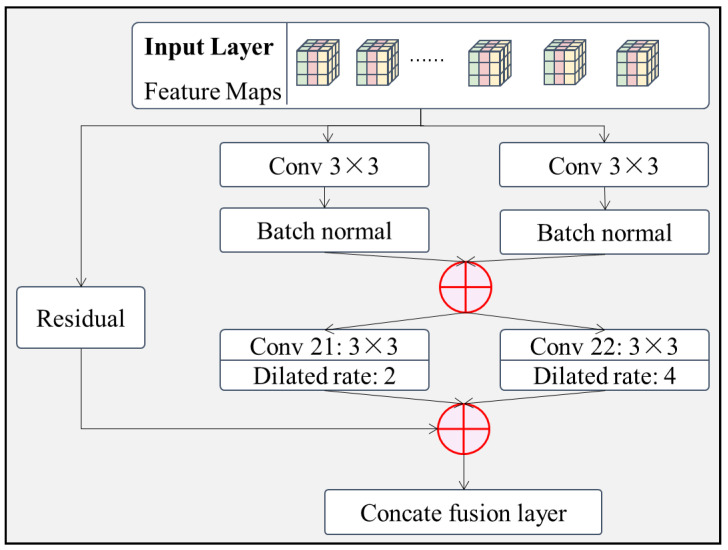
Structure of the residual dilated convolution feature extraction layer.

As data features such as image, audio and vibration mostly satisfy local correlations, the association between features is mainly local and extends from the local to the whole. This type of locally relevant data feature extraction is suitable for the use of RNN (recurrent neural networks) models represented by LSTM (long short-term memory) and others. However, as RNN models cannot be parallelized, they are very time consuming for long sequence data. Therefore, this subsection proposes a residual dilated convolution feature extraction layer for long sequence data by using the feature that CNN models can be parallelized. It also combines different scales of the dilated convolution layer to increase the perceptual field of the feature extraction layer without increasing the network parameters, and extracts feature data at different scales. The module has 2 main branches, as shown in Figure 5.

- Branch 1.
  The original features are passed directly through the intermediate layer without processing and finally fused with the processed feature data by channel connection. This branch is mainly used to prevent overfitting of the model due to too many layers. This branch can also be used to enrich the original features by increasing the number of channels of the original input through a convolutional layer of size 1×1.
- Branch 2.
  Module divides the same input into two 3×3 convolutions and then fuses the results by channel concatenation. Then it is divided in half by two times the number of channels of the previous layer of 3×3 convolution. The expansion rate of convolution kernel 21 is set to two and that of convolution kernel 22 is set to four. Finally, the two dilated convolutions are fused by connecting the channels.

The raw feature data are processed in two different branches and finally fused by the connecting fusion layer by channel to provide the fused feature data for the later model.

(3) A feature adaptive mapping layer.

**Figure 6:**
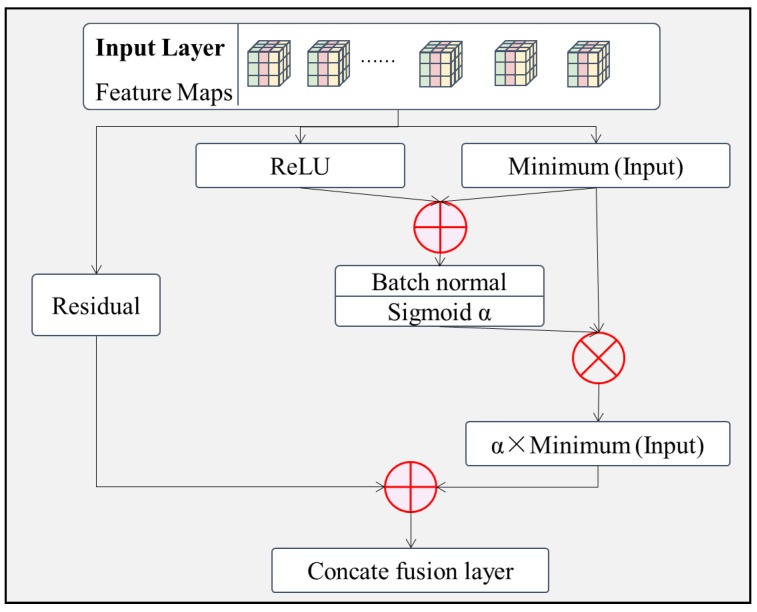
Structure of the feature adaptive mapping layer.

After feature extraction is completed, the neural network uses essentially the exact same non-linear transformation to learn the same type of feature data that was extracted. However, due to the different operations, the differences between the feature data, even under the same operating conditions, can be large. This would make it difficult to classify these feature data into the same category. To address this problem, this subsection introduces a feature adaptive mapping layer that automatically learns to perform non-linear mapping based on the input data features [54]. The module has two main branches, as shown in Figure 6.

- Branch 1.
  The original features are passed directly through the intermediate layers without processing and finally fused with the processed feature data by channel connection. This prevents the model from being overfitted due to the number of layers. Similarly, this branch can be enriched by increasing the number of channels of the original input through a convolutional layer of size 1×1.
- Branch 2.
  The same input is divided into two channels for ReLU (Rectified Linear Unit) regularization and minimization feature extraction [55], where the minimization feature extraction channel is further divided into two channels. The features of the original input ReLU are connected to one of the minimized feature extraction channels and then normalized and sigmoid fused to calculate the α value of the adaptive feature mapping. Finally, α is multiplied with the minimized feature extraction of the other channel to obtain the adaptive feature mapping for that branch.

(4) Time-series data image conversion processing based on the Gramian angular field.

Deep learning techniques are developing rapidly at this stage, especially in the field of imaging, and have made great achievements. For digital signal processing problems, the use of deep neural networks is extremely limited. In computer vision terms, image data is not necessarily a photograph or video, but can be ’image’ data from infrared sensors, accelerometers or other sources. By transforming time series data into images, the advantages of deep learning techniques for image processing can be fully used. In this paper, the Gramian angular field (GAF) algorithm is introduced to convert digitized signals into image data for processing. The Gramian angular field algorithm is suitable for real-time high-frequency signal processing because it can keep the data features uncorrupted while preserving the original time-ordered information [56].

### 3.2. Transfer Learning VGG19 Multilayer Feature Fusion Network

#### 3.2.1. A Transfer Learning VGG19 Multilayer Feature Fusion Network

Transfer learning is a method of learning from a previously similar task and applying it to a new one. The aim is to extract knowledge and experience from one or more source tasks and apply it to a new target domain to reduce the cost of model training [57]. Thanks to transfer learning and deep learning pre-training networks, the problem of training models with a small sample data is well solved. A very popular strategy in current deep learning is to fine-tune the underlying structure of the pre-trained network for the characteristics of the domain dataset [58]. Commonly used pre-trained models are VGG16, VGG19, ResNetV2, InceptionV3, MobileNetV2, DenseNet, NASNet and other classical network architectures. Since the VGG neural network (very deep convolutional networks designed by visual geometry group) is a classical simple linear structured network with high network recognition accuracy and easy modification of its network structure, the VGG network is chosen as the backbone network in this paper [59], compared to VGG19, VGG11, VGG13 and VGG16, which are shallow networks. Due to the small amount of training data of the network in this paper, the recognition accuracy of the network is relatively low, and the convergence of the network is poor on the shallow network. Therefore, this paper freezes the first 15 layers of the VGG19 network without training and uses them as the backbone network.

**Figure 7:**
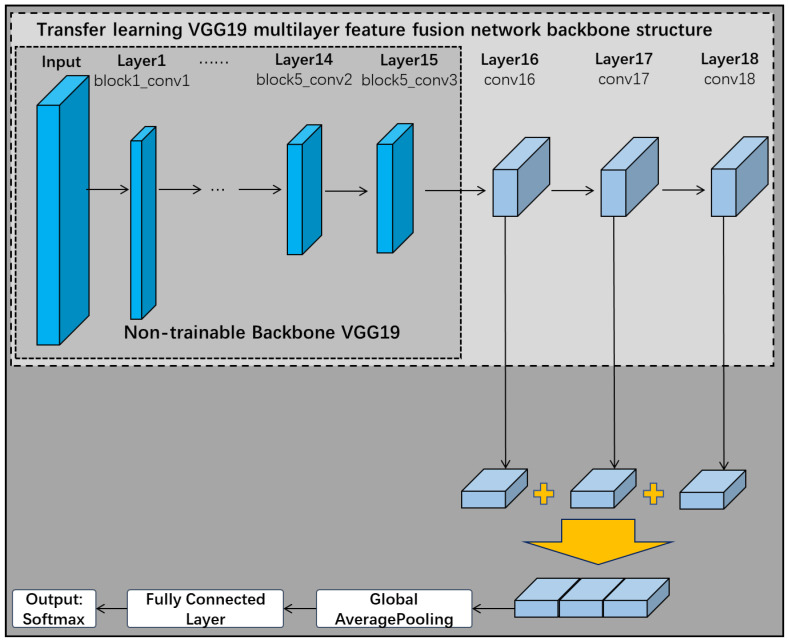
Transfer learning VGG19 multilayer feature fusion network.

As shown in Figure 7, this subsection removes the three fully connected layers of the VGG19 network when choosing it as the backbone of the pre-trained network. The first 15 of these frozen layers remain untrained, but the remaining one convolutional layer and three fully connected layers in the original network are replaced by three new convolutional network layers. The improved VGG19 backbone neural network therefore has a total of 18 layers. These 3 new convolutional network layers are connected in turn, and eventually the multilayer features are fused through a fusion layer. The feature extraction layer designed in Subsection 3.1 can be connected to the frozen layer structure of VGG19 for feature extraction according to demand, and finally completes the construction of the transfer learning VGG19 multilayer feature fusion network.

#### 3.2.2. Model Performance Evaluation Metrics

Model performance and error are closely related and Accuracy, Precision, Recall and F1 measure are ideal metrics for evaluating the performance of various network models. To reflect the advantages and disadvantages of the model, Equations (1) to (4) are used in this paper to measure the performance of the model.

(1) $$Accuracy=\frac{TP+TN}{TP+TN+FP+FN}$$

(2) $$Precision=\frac{TP}{TP+FP}$$

(3) $$Recall=\frac{TP}{TP+FN}$$

(4) $$F1=\frac{2TP}{2TP+FP+FN}$$

where in Equations (1) to (4), TP is the true positive class, FP is the false positive class, FN is the false negative class and TN is the true negative class.

For the problems of long-tailed data distribution and uneven sample identification difficulty caused by small sample data, this section solves such problems well by introducing the focal loss technique [60], as shown in Equation (5).

(5) $$FL\left(p_{t}\right)=-\alpha {\left(1-p_{t}\right)}^{\gamma }log\left(p_{t}\right)$$

where, $p_{t}$ is the probability that the prediction is a true label, γ is the shape of the controlling focal loss curve, and α is the inter-category weighting control factor.

## 4. Improved Entropy-Weighted Topsis Method Multi-Source Data Decision-Level Fusion Evaluation System and Fusion Strategies

### 4.1. Improved Entropy-Weighted Topsis Method Fusion Evaluation System

#### 4.1.1. Topsis Method Fusion Evaluation System

For the construction of the evaluation system, this paper uses the topsis (technique for order preference by similarity to an ideal solution) method. The topsis method is a commonly used comprehensive evaluation method that makes full use of the information in the raw data and accurately reflects the gaps between the evaluation options.Assuming that there are *n* models to be evaluated and *m* evaluation metrics in the topsis evaluation system, where $x_{ij}$ is the value of the index of the *m* evaluation metric of the *n* model to be evaluated. The algorithm flow is as follows.

1. Indicator attributes are positively processed, converting all low and medium performance indicators into high performance metrics.
  (6) $$x_{ij}=\left\{\begin{array}{cc} x_{ij} & \mathrm{High}-\mathrm{performance}\;\mathrm{metric}\\ 1/x_{ij} & \mathrm{Low}-\mathrm{performance}\;\mathrm{metric}\\ M/(M+\left|x_{ij}-M\right|) & \mathrm{Medium}-\mathrm{performance}\;\mathrm{metric} \end{array}\right.$$
2. Normalization of the data to obtain the normalized matrix *Z*.
  (7) $$Z=\left[\begin{array}{cccc} Z_{11} & Z_{12} & \cdots & Z_{1m}\\ Z_{21} & Z_{22} & \cdots & Z_{2m}\\ \cdots & \cdots & \cdots & \cdots \\ Z_{n1} & Z_{n2} & \cdots & Z_{nm} \end{array}\right]$$
3. Determine the optimal and inferior solutions. The optimal solution $Z^{+}$ consists of the maximum value in each column of *Z* and the inferior solution $Z^{-}$ consists of the minimum value in each column of *Z*.
4. Calculate the distances $D_{i}^{+}$ and $D_{i}^{-}$ for each of the evaluation objects from $Z^{+}$ and $Z^{-}$.
  (8) $$D_{i}^{+}=\sqrt{\sum_{i=1}^{m}{\left(maxZ_{ij}-Z_{ij}\right)}^{2}}$$
  (9) $$D_{i}^{-}=\sqrt{\sum_{i=1}^{m}{\left(minZ_{ij}-Z_{ij}\right)}^{2}}$$
5. Calculate the proximity of each evaluation object to the optimal solution $C_{i}$.
  (10) $$C_{i}=\frac{D_{i}^{-}}{D_{i}^{+}+D_{i}^{-}}$$
  where, $C_{i}$ denotes the proximity of each evaluation object to the optimal solution. $0\leq C_{i}\leq 1$, the closer $C_{i}$ is to 1, the better the evaluation object is.

#### 4.1.2. Improved Entropy-Weighted Topsis Method of Fusion Evaluation System

However, the topsis method is established on the basis that the individual evaluation indicators are all equivalent, which is not objective in use. Weights need to be added between the evaluation indicators to reflect the differences between them. There are currently two main methods for increasing the weight between indicators, the analytical hierarchy process (AHP) [61] and the entropy weight method (EWM) [62]. The AHP method mainly draws on the empirical knowledge of domain experts to obtain the weights of the different factors. However, the AHP method is not practical due to the scarcity of experts in the field. Therefore, this paper uses the entropy weighting method to construct the weighting coefficients among the indicators. The process is as follows.

1. Calculate the weight of the *n*th model to be evaluated under the *m* evaluation indicator for that indicator.
  (11) $$p_{ij}=\frac{X_{ij}}{\sum_{i=1}^{n}X_{ij}}$$
  where, i=1,2,⋯,n;j=1,2,⋯,m.
2. Calculate the entropy value of indicator *j*.
  (12) $$k=\frac{1}{\ln(n)}$$
  (13) $$e_{j}=-k\sum_{i=1}^{n}p_{ij}\ln\left(p_{ij}\right)$$
  where, $e_{j}\geq 0$.
3. Calculation of information entropy redundancy (degree of dispersion).
  (14) $$d_{j}=1-e_{j}$$
4. The final entropy weight values for each indicator are obtained as follows.
  (15) $$w_{j}=\frac{d_{j}}{\sum_{j=1}^{m}d_{j}}$$
5. In order to measure the contribution of each model before fusion, this paper specifically establishes the contribution percentage $P_{i}$, which is calculated for each model before fusion according to Equation (16) as follows.
  (16) $$P_{i}=\frac{C_{i}}{\sum_{i=1}^{n}C_{i}}\times 100\%$$
  where, the contribution of each independent model $P_{i}\geq 1/n$ is a valid model that can be fused.

The improved fusion evaluation system screens for differences in the performance of models on different data sources and removes poor performing models from the fusion system when they appear. Only the models with better performance are retained as the final fusion models. This not only reduces the number of models to be fused and reduces computational effort, but also improves generalization accuracy and model comprehensibility, ultimately improving fusion performance.

### 4.2. Decision-Level Fusion Algorithms and Fusion Strategies for Multi-Source Data

#### 4.2.1. Multi-Source Data Fusion Based on Adaboost Algorithm

For the model fusion of multi-source data, the augmentation method strategy of integrated learning assigning different weights to different sub-models has some reference value. The multi-source data fusion in this section is based on the AdaBoost algorithm and combines its findings with multi-source data fusion. The main idea of the AdaBoost algorithm is to assign higher weights to classifiers with high accuracy, and eventually multiple classifiers are weighted by a weighted set to become a strong classifier with high accuracy [63]. The algorithm flow is as follows.

1. For the training dataset $D_{0}=\left\{\left(x_{i},y_{i}\right)|i=1,\cdots ,N\right\},y_{i}\in \left\{-1,+1\right\}$, the weak learning algorithm *L* trains a total of *R* iterations, where the training samples are all given the same weight 1/N at the beginning.
  (17) $$D_{1}=\left(w_{11},w_{12},\cdots ,w_{1N}\right)$$
2. Several iterations were performed to learn using a training dataset with a weight distribution $D_{R}$ to obtain the weak classifier $G_{R}\left(x\right)$.
  (18) $$G_{R}\left(x\right)=X\to \left\{-1,+1\right\}$$
  where $D_{R}$ represents the probability distribution (or weight distribution) of the training data before the start of the *R*th iteration.
3. Calculate the classification error rate of $G_{R}\left(x\right)$ on the training dataset.
  (19) $$e_{R}=P\left(G_{R}\left(x_{i}\right)\neq y_{i}\right)=\sum_{i=1}^{N}w_{Ri}L\left(G_{R}\left(x_{i}\right)\neq y_{i}\right)$$
  where $w_{Ri}$ denotes the weight at the *i* sample.
4. Calculate the coefficients of the weak classifier $G_{R}\left(x\right)$.
  (20) $$a_{R}=\frac{1}{2}log\frac{1-e_{R}}{e_{R}}$$
  where the smaller the classification error rate, the greater the role of the weak classifier in the final classifier.
5. Updating the weight distribution of the training dataset for the next iteration.
  (21) $$D_{R+1}=\left(w_{R+1,1},w_{R+1,2},\cdots ,w_{R+1,N}\right)$$
  (22) $$w_{R+1,i}=\frac{w_{Ri}}{Z_{R}}exp\left(-a_{R}y_{i}G_{R}\left(x\right)\right)$$
6. Combination of individual weak classifiers.
  (23) $$f\left(x\right)=\sum_{i=1}^{R}a_{R}G_{R}\left(x\right)$$
7. To obtain the final strong classifier.
  (24) $$G\left(x\right)=sign\left(f\left(x\right)\right)=sign\left(\sum_{i=1}^{R}a_{R}G_{R}\left(x\right)\right)$$

However, the AdaBoost algorithm is sensitive to noisy data, which can lead to disadvantages such as higher weights in iterations and more time-consuming fusion training, affecting its final performance. These problems also create challenges for the fusion of multi-source data and require improvements to their fusion strategies.

#### 4.2.2. Improved Evaluation Indicators and Integration Strategies

(1) Improved evaluation indicators.

In general, accuracy, precision, recall and F1 values are ideal metrics for evaluating the performance of various classification algorithms. However, for the multi-source data fusion process, these evaluation metrics are not indicative of performance changes in model fusion and can only determine the relative performance of the model. The evaluation metrics need to be improved. Since this paper splits the multi-classification problem into several binary classification problems, the category coding of the classification targets is using the one-hot coding method. The confidence level for each category is a probability matrix within the interval [0,1] calculated by the softmax function. The more certain the model is that the prediction is correct for a given input, then the closer its confidence value is to 1. Therefore, the evaluation index in this paper still retains the dimensionless evaluation index accuracy, and at the same time, introduces a new dimensionless evaluation index confidence $P_{c}$ for each category as an evaluation index, as shown in Equation (25).

(25) $$P_{c}\left(i,j\right)=softmax\left(y_{i}^{\prime }\right)=\frac{e^{y_{i}^{\prime }}}{\sum_{j=1}^{K}e^{y_{i}^{\prime }}}$$

where $\sum_{j=1}^{K}P_{c}\left(i,j\right)=1$, $y_{i}^{\prime }$ is the output of the corresponding category of the model. *i* is the predicted object, *j* is the *j*th element of the predicted object, and *K* is the number of categories of the multi-classification problem.

For the evaluation system consisting of accuracy and $P_{c}$ indicators, accuracy and $P_{c}{\left(j,i\right)}_{max}$ at the location of element 1 are high performance indicators, and the remaining indicators are low performance indicators. In order to simplify the rating system and reduce the number of evaluation indicators, the fusion system of multi-source data in this paper is judged by high superiority indicators, including one accuracy indicator and the confidence evaluation indicator $P_{c}{\left(j,i\right)}_{max}$ for *K* classification categories. The prediction accuracy of the model for each category is closely related to the confidence evaluation indicator $P_{c}$. The current model prediction is mainly to rank the confidence $P_{c}$ of each position and take out the category corresponding to the maximum confidence to complete the prediction. However, for anomalous or noisy data, the model will have low confidence in its prediction, resulting in poor robustness and generalization of the trained model.

To address this issue, this section sets a threshold value for the confidence evaluation indicator $P_{c}$. The maximum confidence level for the sample exceeds the threshold, and the prediction is correct before it is considered a valid prediction, which is recorded in the classification accuracy. If the maximum confidence level of a sample exceeds the threshold but the category is incorrectly predicted, it is considered to be an invalid prediction, the prediction is not recorded in the accuracy, and its confidence level $P_{c}$ is reset to 0, as shown in Equation (26).

(26) $$P_{c}{\left(j,i\right)}_{max}=\left\{\begin{array}{c} P_{c}{\left(j,i\right)}_{max},Threshold\leq P_{c}{\left(j,i\right)}_{max}andPredict_{label}=True_{label}\\ 0,otherwise \end{array}\right.$$

The problem with setting the threshold value is that taking too high a value will result in the model not converging. Conversely, low prediction accuracy and poor performance of the model will occur. In this paper, the multi-classification problem is split into multiple binary classification problems, so that the $P_{c}{\left(j,i\right)}_{max}$ is only slightly higher than the accuracy of 0.5 for random guesses. Therefore, the threshold value is set to 0.5.

(2) Dynamic fusion strategies.

For the fusion integration of several data sources and models, the inference time increases exponentially with the number of fused models. Selecting after each iteration use the fusion evaluation system would be extremely costly in terms of computing power and time, and would result in incorrect selection due to some anomalous data. To address this problem, the dynamic fusion strategy in this subsection calculates the accuracy, the mean confidence $P_{c}{\left(j,i\right)}_{max}$ for each category, every 50 iterations. When a model has a contribution $P_{i}\leq 1/n$ for 2 consecutive times (i.e., 100 rounds iterations), this indicates that the model on that data source impairs the performance of the fusion model and it is removed from the fusion system. The fusion of models on the remaining data sources is continued, with the final fused model n≥3.

**Figure 8:**
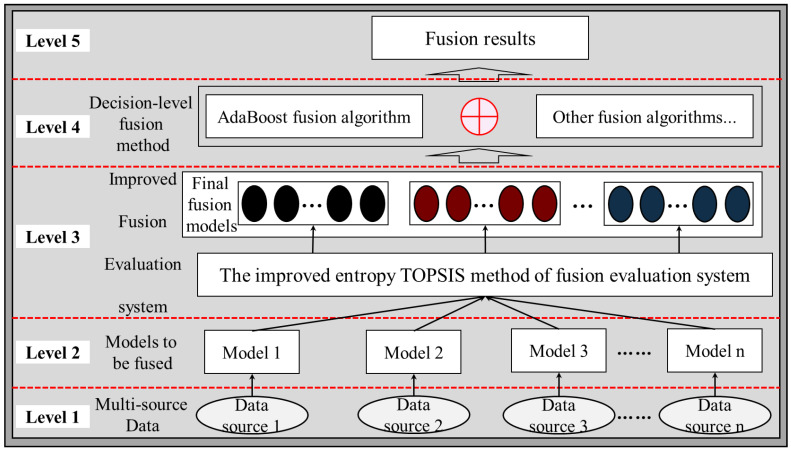
Multi-source data fusion evaluation system and fusion strategiesr.

As shown in Figure 8, the process of fusing multi-source data evaluation system and fusion strategies to select quality models is demonstrated. This multi-source data fusion evaluation system has few evaluation metrics, each evaluation metric is dimensionless, and the use of filtered high-quality models for fusion can effectively alleviate the shortcomings of multi-source data fusion in terms of data source quality, model quality, and training inference time.

## 5. Experimental Tool Wear State Diagnosis and Comparative Algorithm Performance Analysis

### 5.1. Experimental Environment, Data Preparation and Validation Process

#### 5.1.1. Experimental Environment

The algorithm in this paper runs on a Windows 7 PC with an I5-8500 processor, NVIDIA GTX1050 graphics card with 4G capacity, 32G of RAM and 1T of hard disk. The algorithm programming is implemented using the Python 3.6 programming language, Keras and the TensorFlow 1.15 framework. End mills dry cut 45 steel workpieces with dimensions of 100 mm × 100 mm × 20 mm under the condition of no coolant. Four-flute carbide milling cutters with an 8 mm cutting edge, 24 mm cutting edge length and 60 mm total length are used. The vibration signal is acquired by a WB1550T sensor and the milling force signal is acquired by a Kistler 9257b sensor, with a signal acquisition frequency of 20 KHz for each channel. The spindle speed of the machine is 2000 rpm, the feed rate in X-axis direction is 800 mm/min, the cutting width is 8 mm, and the cutting depth is 0.2 mm. Each milling 80 mm is recorded as one tool walk and the VB value of the back tool face wear is measured, totaling 300 cycles of tool walks.

#### 5.1.2. Data Preparation

**Figure 9:**
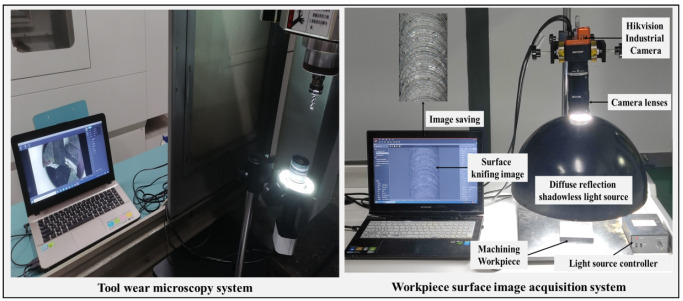
Tool wear measurement system and surface image acquisition system.

In this paper, the mean value of VB = 0.12 mm for the wear width of the back face of the end mill is used as the dulling standard. Among them, 0.07 mm or less is the initial wear, 0.07–0.12 mm is normal wear, and more than 0.12 mm is a rapid wear. A total of 43 times of initial wear, 179 times of normal wear, 37 times of rapid wear, and 41 times of abnormal wear vibration were collected. For the diagnostic identification of the state of the tool, this paper collects the milling force signal of X/Y/Z axis, the vibration signal of X/Y/Z axis, the image of the knife mark of the workpiece after machining, a total of 7 data sources data. As shown in Figure 9, the tool wear value VB is measured using a tool microscope with 500 times magnification, and the workpiece surface knife mark is captured by an image acquisition system consisting of a high-definition industrial camera and a diffuse reflection no-shadow light source.

**Figure 10:**
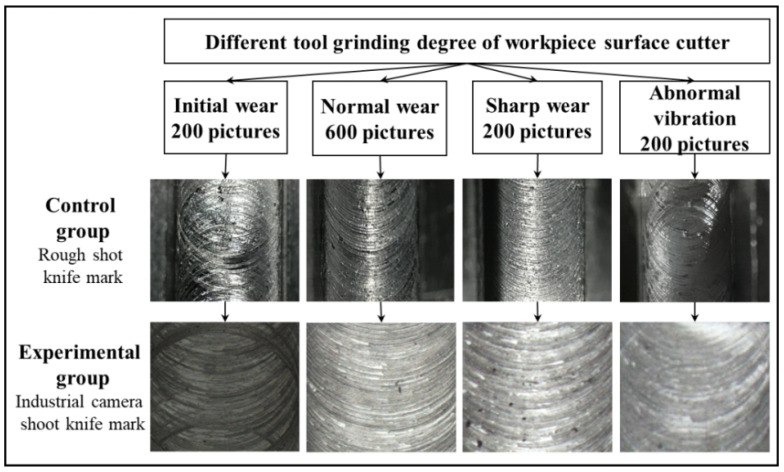
Workpiece surface knife mark in four states.

As shown in Figure 10, the tool surface image dataset has a significant small sample with long-tail distribution problem. In the normal wear state, 600 grayscale images of size 200×200 pixels were acquired, and 200 grayscale images of size 200×200 pixels were acquired in each of the other three states, for a total of 1200 images.

#### 5.1.3. Experimental Validation Process

The experiments in this chapter are divided into experimental validation of multilayer feature fusion on a single data source and decision-level fusion on multi-source data. Experiments on a single data source are subdivided into two parts. Part one, Max-AVG pooling multilayer feature fusion network in Section 5.2.1 tests its performance on knife mark image data. Part two, improved transfer learning VGG19 multilayer feature fusion network in Section 5.2.2 tests its performance on the milling force signal. The experiments on multi-source data in Section 5.3 are decision-level fusion experiments on seven data sources, including milling forces in the X/Y/Z axes, vibrations in the X/Y/Z axes, and knife mark images. The aim of the experiments is first to verify the performance of the multilayer feature fusion networks proposed in this paper on a single data source. Then, the multi-source data fusion evaluation system and fusion strategies proposed in this paper are validated. Finally, the fusion method proposed in this paper is compared with the current mainstream multi-source data fusion methods to verify the advantages and disadvantages of our method.

### 5.2. Single-Source Data for Tool Wear Status Diagnosis

#### 5.2.1. Max-AVG Pooling Multilevel Feature Fusion Tool Wear Status Diagnosis

(1) Transfer learning VGG19 multilayer feature fusion network.

This subsection uses the transfer learning VGG19 multilayer feature fusion network structure in Section 3.2.1. The learning rate of the network is set to $1\times 10^{-6}$, the decay momentum is set to 0.9, and the number of training iteration epochs is set to 500. The experiments were conducted with 1200 images of knife mark samples for four wear status of tools, of which 80% were used as training data. Since the data set has the problem of small samples with long-tailed distribution, and the normal wear produces more knife mark images than the rest of the status, 40 images were taken as test samples for all four statuses in order to prevent the overall test accuracy of the model from being affected during the test.

**Figure 11:**
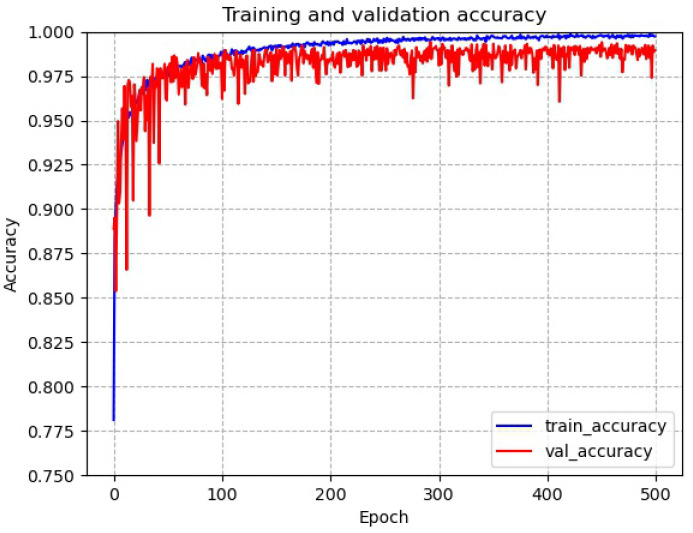
Accuracy of the network for tool wear status diagnosis.

Figure 11 shows the accuracy of the network for tool wear status diagnosis. It can be seen that the network has a serious overfitting problem on the training dataset. The network oscillates strongly on the test dataset in the early stages of training, and there is a sudden drop in accuracy in the middle and late stages. This indicates that the network structure is not well designed for datasets with small samples with long-tailed distribution problems and needs to be improved.

(2) Max-AVG pooling multilayer feature fusion network for tool wear diagnosis.

**Figure 12:**
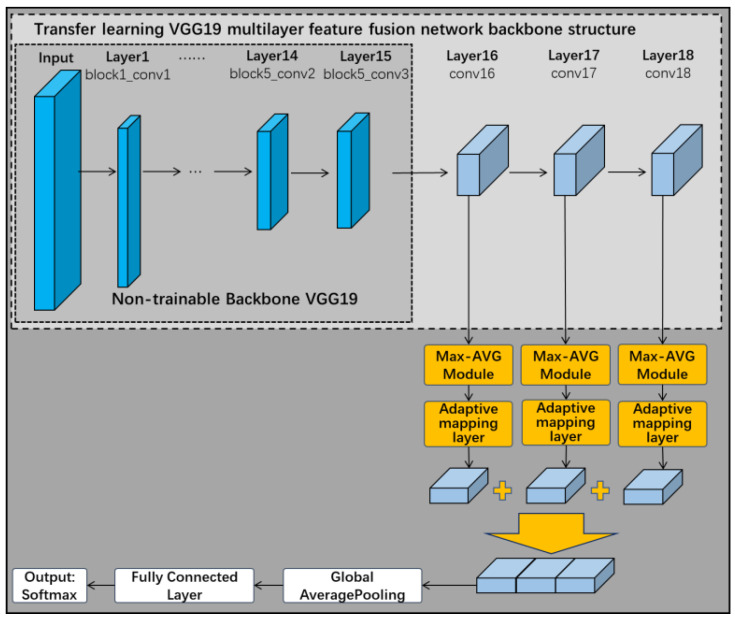
Max-AVG pooling multilayer feature fusion network.

Considering the characteristics of the target surface area to be detected, the Max-AVG pooling feature extraction layer and the feature adaptive mapping layer from Section 3.1 are combined with the transfer learning VGG19 multilayer feature fusion network. As shown in Figure 12, the Max-AVG pooling feature extraction layer and the feature adaptive mapping layer are connected after the last three trainable convolutional layers to complete the feature extraction and feature adaptive mapping, respectively. The feature adaptive mapping layer contains a residual channel, which not only enables adaptive mapping of the extracted features, but also prevents overfitting.

**Figure 13:**
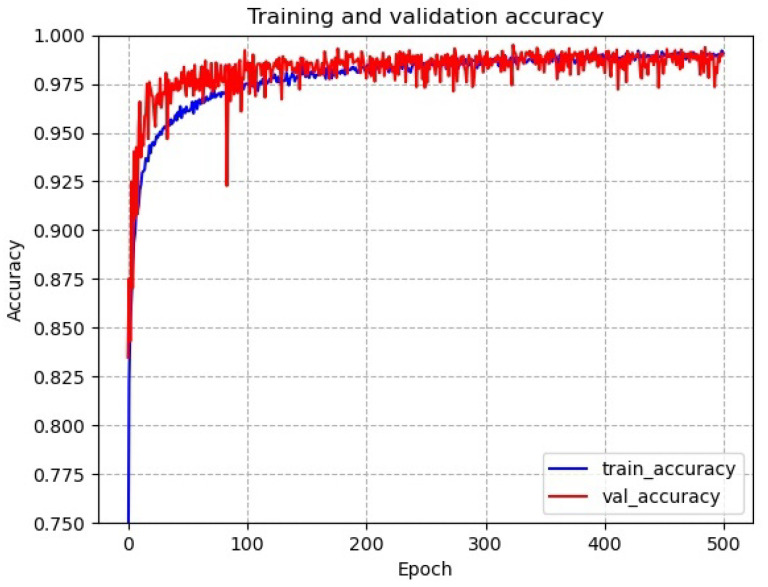
Accuracy of Max-AVG pooling network for tool wear condition diagnosis.

As shown in Figure 13, the convergence and accuracy of the Max-AVG pooling multilayer feature fusion network is greatly improved from the original network. The training accuracy and test accuracy of the network converged as the network training progressed, and the network did not suffer from overfitting. After experimental testing, the final accuracy of the model eventually converged at around 97.0% (±0.5% variation).

(3) Performance comparison of two improved network models.

As it can be seen from Table 2, the improved Max-AVG pooling network model has a significantly higher recall rate for the rapid wear status than before the improvement. However, the recall rate for the two statuses of normal wear and abnormal vibration wear is slightly lower than that before the improvement. As there are significantly more images of normal wear than the remaining three states of the knife mark, the surface knife mark morphology is more distinct, so both networks have a high accuracy rate for normal wear. The sharp oxide surface at the early stage of new tool wear is similar to the tool wear that occurs during rapid tool wear, so the identification errors of the two networks are mainly concentrated on the two states of initial wear and rapid wear.

The Max-AVG pooling multilayer feature fusion network outperformed the pre-modified network in all metrics, and effectively suppressed the overfitting problem of small samples and long-tailed distribution data sets.

#### 5.2.2. Improved Residual Dilated Convolution Multilayer Feature Fusion Tool Wear Condition Diagnosis

(1) Improved transfer learning VGG19 multilayer feature fusion network.

**Figure 14:**
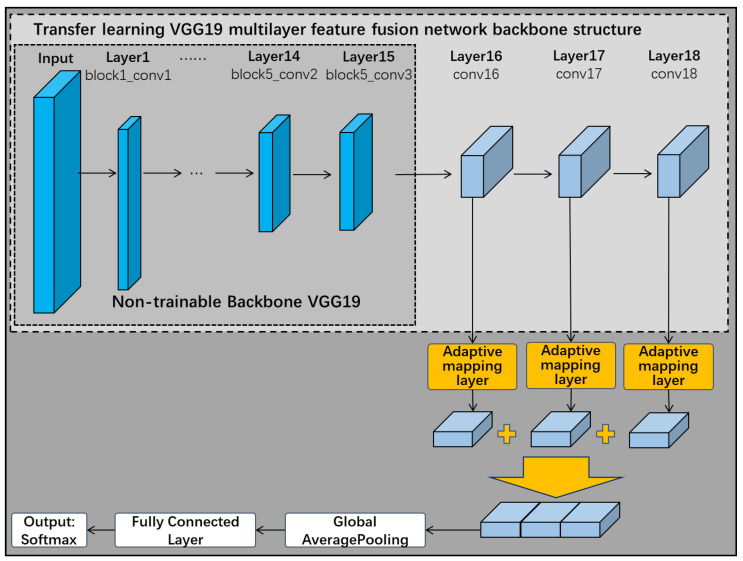
Improved transfer learning VGG19 multilayer feature fusion network.

In this section, we use the Gramian angular field algorithm to convert the milling force signals collected in four tool wear status into images, and continue to diagnose the tool wear status using deep learning techniques. As the milling force signal is collected at a frequency of 20 KHz, the spindle speed is 2000 rpm which is 0.03 sec/rev. The sensor is capable of collecting approximately 600 force signal points during one cycle of spindle rotation. Taking into account various noise interference factors, the sampling points of the data in this subsection are taken as 600, 900, 1200 and 1800, respectively, to alleviate the model overfitting problem through multi-scale data generation. In Figure 14, this subsection continues to use the transfer learning VGG19 multilayer feature fusion network structure, and improves it by connecting a feature adaptive mapping layer to each of the last three convolutional layers to finalize the multilayer feature fusion. Since the Gramian angular field image of the milling force signal does not have any obvious defective regions and all regions in the image are valid milling force signals, the Max-AVG pooling feature extraction layer is removed from the tool wear status diagnostic network in this subsection.

The learning rate of the network is set to 0.01, the decay momentum is set to 0.9 and the number of training iterations is set to 750 epochs. A total of 36,000 sample images are generated for each of the four tool states, of which 80% are used as training data and 20% as test data.

**Figure 15:**
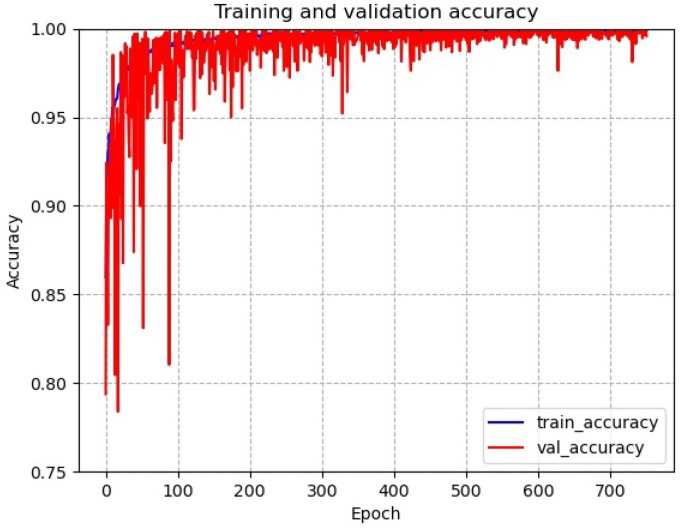
Accuracy of improved network for tool wear status diagnosis.

Figure 15 shows the accuracy of the improved transfer learning VGG19 multilayer feature fusion network, and the curve of the network accuracy shows that the model has overfitting problems during the training process. During the test, the test recognition accuracy did not converge, and even showed a significant decrease. As the Gramian angular field image of the signal does not have obvious defect areas, the four tool statuses are relatively homogeneous and rich in features, although the amount of image data is high. The method of continuing to increase the depth of the network introduces serious overfitting problems and is not suitable for this type of image, which requires improvements to the feature extraction layer.

(2) Improved residual dilated convolution multilayer feature fusion network for tool wear diagnosis.

**Figure 16:**
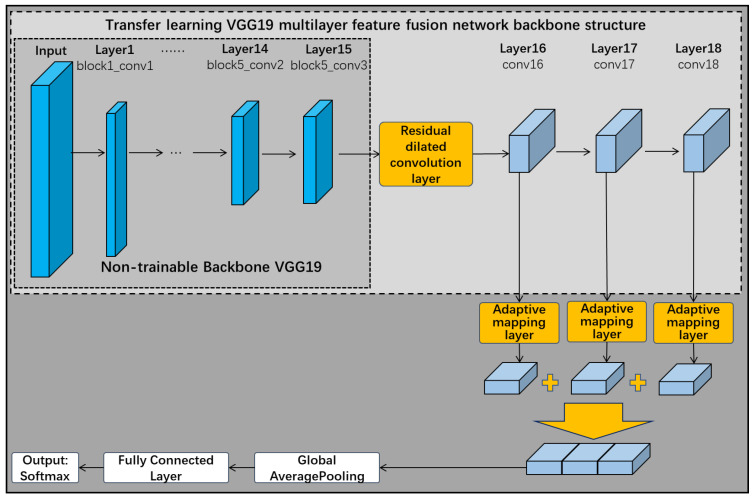
Improved residual dilated convolution multilayer feature fusion network.

For the above problem, the residually connected dilated convolutional feature extraction layer in Section 3.1 is combined with the VGG19 multilayer data feature fusion network. The dilated convolution layer at different scales increases the perceptual field of the feature extraction layer without increasing the network parameters, and extracts feature data at different scales. Due to the high number of convolutional kernels and network layers of the residual dilated convolutional layer module, and the inclusion of a residual connected channel with a high number of parameters, adding a residual dilated convolution module before each adaptive mapping layer would increase the computational effort of the network and make it more difficult to train and converge. Considering the unstable accuracy of the original diagnostic network during testing, the residual dilated convolutional layer module is added after the VGG19 network backbone layer, where the network parameters are not trainable. Figure 16 shows that this not only utilizes the ability of the residual connectivity module to suppress overfitting, but also increases the perceptual field of the feature extraction layer and reduces the computational effort and training parameters of the network.

**Figure 17:**
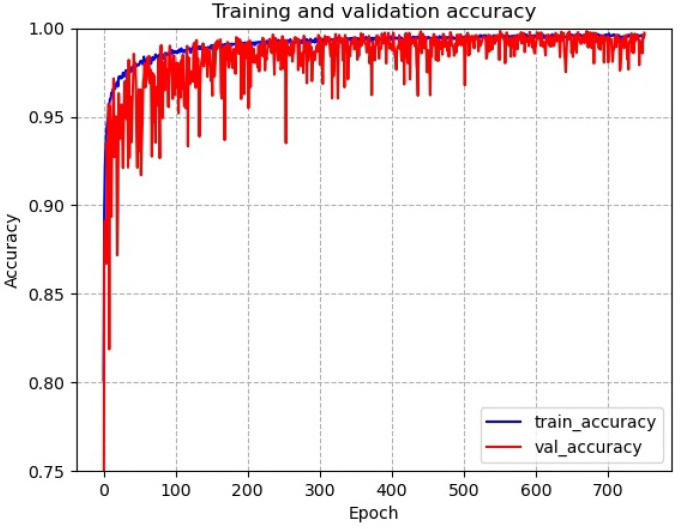
Accuracy of improved residual dilated convolution multilayer feature fusion network.

Figure 17 shows the accuracy and loss error of the improved residual dilated convolution multilayer feature fusion network. It can be seen that the improved network effectively alleviates the model overfitting problem during the training process, and the stability has been greatly improved. The test accuracy of the model finally converged at around 98.1% (±0.5% fluctuation). The training speed of the improved network is a little reduced compared to the original network due to the addition of residual channels after the backbone layer of the untrainable VGG19 network.

(3) Performance comparison of two improved network models.

As it can be seen in Table 3, the improved network model has a higher recall than the VGG19 multilevel diagnostic network for the two statuses of normal wear and vibration abnormal wear, but a slightly lower recall for the two statuses of initial wear and rapid wear.

The improved transfer learning VGG19 multilayer feature fusion network suffered from overfitting problems during the training process. Although the improved residual dilated convolution multilayer feature fusion network is slightly lower in performance than the pre-improvement network, the stability and convergence of the model are better. This is even more practical for raw signal data containing complex noise.

### 5.3. Multi-Source Data Fusion for Tool Wear Status Diagnosis

(1) Simplified VGG19 multilayer feature fusion network.

**Figure 18:**
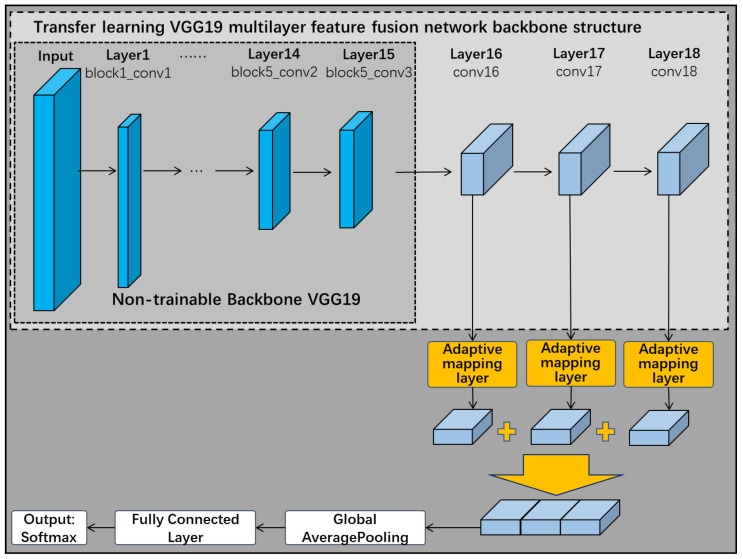
VGG19 multilayer feature fusion network for multi-source data fusion.

This subsection of the experiment introduces seven data sources, such as milling forces in the X/Y/Z axes, vibrations in the X/Y/Z axes and images of surface knife mark. To facilitate feature extraction and the fusion of sensor data by deep neural networks, the Gramian angular field algorithm is used to transform the sensor signal data. A total of four tool wear statuses for each of the six sensor sources generate 9000 sample images, for a total of 6×4×9000 sample images. As shown in Figure 18, the network structure used in this chapter for multi-source data fusion is still a transfer learning VGG19 multilayer feature fusion network, retaining only the adaptive feature mapping layer to prevent overfitting problems during network training, and not using a specially designed feature extraction layer. This not only simplifies the structural design of the model, but also reduces the difficulty of training and deployment.

(2) Comprehensive evaluation of the performance of the dynamic fusion process of multi-source data.

The multi-source data fusion is mainly designed to solve the four tool state diagnostics with five metrics: the accuracy of the test data and the average of the four confidence levels $P_{c}{\left(j,i\right)}_{max}$ for the four states after every 50 epochs. Error metrics are calculated every 50 epochs to select model data sources that can be fused. The evaluation is then carried out through a modified entropy-weighted topsis method fusion evaluation system. If a data source appears to have a contribution $P_{i}$ less than 1/n of the current data source to be fused for 2 consecutive times (i.e., 100 consecutive iterations), it is removed from the fusion system and the fusion filtering continues for the remaining data sources. A comprehensive evaluation of the performance of the dynamic fusion process of the model is shown in Table 4.

As can be seen from the table above, the percentage contribution $P_{i}$ of the three data sources, Y-axis milling force, Z-axis milling force and Z-axis vibration, in epochs 1 to 100 is lower than 1/7 of the seven data sources currently to be fused, so they are removed from the fusion process. Similarly, in epochs 101 to 200, among the remaining four model data sources to be fused, the Y-axis vibration data source with a contribution percentage $P_{i}$ below 1/4 was removed from the fusion process. In epochs 201 to 300, three model data sources remain for the X-axis milling forces, X-axis vibration, and knife mark. So the final fusion model is a model fusion of the VGG19 network on the X-axis milling force, X-axis vibration, and knife mark.

### 5.4. Performance Comparison of Tool Wear Status Diagnostic Networks

**Figure 19:**
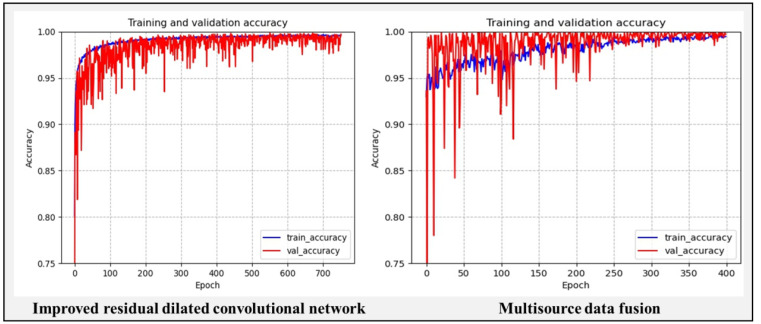
Comparison of the accuracy and convergence performance of the two networks.

In Figure 19, to demonstrate the performance of multi-source data fusion, the performance is compared with that of the improved residual dilated convolution multilayer feature fusion network before simplification. As can be seen from the performance comparison graph, the test accuracy of the multi-source data fusion finally converged at around 99.2% (±0.5% variation), which has a significant improvement in the stability and accuracy compared to the modified residual dilated convolution network with a single data source before simplification. The performance of the models is unstable in the first 100 epochs due to the use of all seven data sources. The stability and accuracy of the multi-source data fusion model are much worse than the performance of the model on a single data source before simplification. In the subsequent epochs 101 to 200, the evaluation system removes three data sources, such as Y-axis milling force, Z-axis milling force and Z-axis vibration, and the performance of the fusion model is improved greatly, but there are still large fluctuations. This indicates that there is still more noise and abnormal data in the remaining four data sources. In epochs 101 to 200 iterations, the performance of the Y-axis vibration data source for 100 consecutive iterations has a contribution $P_{i}$ less than 1/4 of the model to be fused, so it is removed from the fusion model. In epochs 201 to 300, the multi-source data fusion network finally fused the three data sources, including X-axis milling force, X-axis vibration and knife mark, and the model converged steadily after 250 epochs. To further validate the stability and convergence of the fusion model, the model continued for 100 epochs to test its stability. The stable performance of the fusion model over 301 to 400 epochs further demonstrates that the accuracy and stability of the fusion network is better than that of the single data source model.

As it can be seen in Table 5, the recall of the multi-source data fusion is slightly lower than the performance of the single source model before simplification, except for the vibration abnormal wear status. In the rest of the statuses, the converged network has a significant performance advantage. This is of more practical value for raw signal data containing complex noise, and verifies that the fusion evaluation system for multi-source data proposed in this paper is feasible.

To further validate the effectiveness of the improved fusion evaluation system and fusion strategies, this paper tests them compared with the current mainstream multi-source data fusion algorithms [33,34,35]. The accuracy and real-time detection performance of the algorithms are compared as shown in Table 6. However, as the collaborative architecture requires the construction of individually running models on each data source for fusion, its single average inference time is longer than that of the fusion method of the joint architecture. In contrast, this paper adds a fusion evaluation system to the fusion algorithm, and removes data sources that impair the performance of the fusion model from the fusion system. So the fusion algorithm in this paper finally fuses three data sources with a single average inference time close to that of the joint architecture fusion method and significantly shorter than that of the collaborative architecture fusion method.

In summary, the following can be concluded.

- Simplification of each independent VGG19 multilayer feature network without the use of specially designed extraction layers can reduce the difficulty of model structure design and training deployment.
- The collaborative architecture fusion method has significantly higher accuracy than the joint architecture fusion method and is more suitable for constructing fusion models for multi-source data, but its average single inference time is longer.
- Screening after each iteration using the fusion evaluation system not only takes too long to train the network, but also causes screening errors due to anomalous data. A dynamic fusion strategy is added to the fusion evaluation system to remove data sources that impair the performance of the fusion model. This effectively alleviates the shortcomings of the fusion model in terms of data source quality, model quality, and training inference time.

## 6. Conclusions

To address the problem of inconsistent data scales among multi-source data, which makes the established models difficult to fuse and low fusion accuracy, an improved fusion evaluation system is added to the decision-level fusion algorithm, and a dynamic fusion strategy is proposed. The improved fusion evaluation system integrates the dynamic fusion of model performance on different data sources, making the fusion model insensitive to hyper parameter selection and improving the accuracy and robustness of the fusion model, and obtaining optimal fusion results. The effectiveness of this paper’s improved entropy-weighted topsis method of comprehensive evaluation system is subsequently verified experimentally on single-source data and multi-source data respectively. Compared with the current mainstream multi-source data fusion methods, the multi-source data fusion system and fusion strategy proposed in this paper do not use specially designed feature extraction networks, the model training deployment is less difficult, and the training inference time is shorter. Our fusion method has better performance and can be well adapted to the characteristics of industrial data.

However, there are still some limitations in this paper. Although some results are achieved in this paper, the paper focuses on validating the application of multilayer feature fusion in production through a typical VGG19 network and its modifications. In the study of decision-level fusion, this paper mainly considers the performance of fusion from an algorithmic point of view. Further research is needed on the distribution of raw data and quality improvement issues on data collection, which are also the direction of subsequent research in this paper.

## Figures and Tables

**Table 1:** A comparison of the advantages and disadvantages of different fusion architecture.

Fusion Architecture	Fusion Difficulty	Modelling Difficulty	Data Quality	Generality of Models
Joint structure	Low	Low	High	Strong
Collaborative architecture	Middle	Middle	Low	General
Encoder and decoder architecture	High	High	Middle	Weak

**Table 2:** Performance comparison of the two network models on the test dataset.

	Indicators	Precision	Recall	F1	Number of Samples
Status					
Transfer learning VGG19 multilayer feature fusion network					
Initial		0.9689	0.9601	0.9644	40
Normal		1.0000	0.9946	0.9973	40
Rapid		0.9596	0.9089	0.9336	40
Vibration		0.9319	0.9945	0.9622	40
Weighted average		0.9652	0.9646	0.9645	160
Max-AVG pooling multi-layer feature fusion network					
Initial		0.9706	0.9601	0.9653	40
Normal		1.0000	0.9910	0.9955	40
Rapid		0.9519	0.9362	0.9440	40
Vibration		0.9561	0.9909	0.9732	40
Weighted average		0.9697	0.9696	0.9696	160

**Table 3:** Performance comparison of the two network models on the test dataset.

	Indicators	Precision	Recall	F1	Number of Samples
Status					
Improved transfer learning VGG19 multilayer feature fusion network					
Initial		0.9544	0.9905	0.9721	1797
Normal		0.9994	0.9649	0.9819	1796
Rapid		0.9888	0.9855	0.9872	1795
Vibration		0.9978	0.9978	0.9978	1795
Weighted average		0.9851	0.9847	0.9847	7183
Improved residual dilated convolution multilayer feature fusion network					
Initial		0.9710	0.9699	0.9705	1797
Normal		1.0000	0.9761	0.9879	1796
Rapid		0.9696	0.9772	0.9734	1795
Vibration		0.9825	0.9994	0.9909	1795
Weighted average		0.9808	0.9806	0.9807	7183

**Table 4:** Dynamic fusion process performance evaluation of multi-source data.

Iterations 1 to 50 epochs, n = 7, 1/7 × 100% = 14.29%							
Models to be fused, n = 7	X-axis force	Y-axis force	Z-axis force	X-axis vibration	Y-axis vibration	Z-axis vibration	Knife mark
Average initial $P_{c}$	0.93553	0.50137	0.50055	0.72734	0.54188	0.64742	0.91871
Average normal $P_{c}$	0.95068	0.53221	0.20076	0.88551	0.64041	0.23609	0.96354
Average rapid $P_{c}$	0.83053	0.55883	0.53731	0.45962	0.70842	0.00548	0.69315
Average vibration $P_{c}$	0.92077	0.70354	0.52611	0.83515	0.88864	0.56858	0.94214
Accuracy	0.93886	0.80122	0.72632	0.79387	0.87563	0.73872	0.91226
$P_{i}$	23.05%	13.31%	10.29%	15.01%	16.72%	1.35%	20.26%
Iterations 51 to 100 epochs, n = 7, 1/7 × 100% = 14.29%							
Models to be fused, n = 7	X-axis force	Y-axis force	Z-axis force	X-axis vibration	Y-axis vibration	Z-axis vibration	Knife mark
Average initial $P_{c}$	0.95327	0.51935	0.52537	0.68532	0.54533	0.73396	0.89905
Average normal $P_{c}$	0.98794	0.55006	0.29086	0.91657	0.68012	0.24012	0.98915
Average rapid $P_{c}$	0.79703	0.56137	0.41454	0.63153	0.71289	0.01458	0.82687
Average vibration $P_{c}$	0.98111	0.71181	0.53182	0.83876	0.86317	0.53549	0.96732
Accuracy	0.94666	0.82621	0.76755	0.82646	0.87911	0.74805	0.94345
$P_{i}$	21.70%	12.77%	8.35%	17.33%	16.27%	1.65%	21.93%
Remove Y/Z axis milling forces, Z-axis vibration							
Iterations 101 to 150 epochs, n = 4, 1/4 × 100% = 25.00%							
Models to be fused, n = 7	X-axis force			X-axis vibration	Y-axis vibration		Knife mark
Average initial $P_{c}$	0.97794			0.89208	0.59352		0.93115
Average normal $P_{c}$	0.93789			0.83802	0.68362		0.97015
Average rapid $P_{c}$	0.73433			0.68753	0.70032		0.80446
Average vibration $P_{c}$	0.98949			0.78541	0.84709		0.94289
Accuracy	0.93412			0.88805	0.85415		0.93997
$P_{i}$	36.52%			25.95%	2.46%		35.06%
Iterations 151 to 200 epochs, n = 4, 1/4 × 100% = 25.00%							
Models to be fused, n = 7	X-axis force			X-axis vibration	Y-axis vibration		Knife mark
Average initial $P_{c}$	0.94773			0.88617	0.74699		0.84693
Average normal $P_{c}$	0.94805			0.92158	0.64221		0.98643
Average rapid $P_{c}$	0.88393			0.72661	0.84277		0.89975
Average vibration $P_{c}$	0.95157			0.93692	0.88595		0.97466
Accuracy	0.96616			0.91627	0.85883		0.93677
$P_{i}$	34.21%			27.10%	5.58%		33.11%
Remove the Y-axis vibration and the final fused data source is 3. Iterations 201 to 300 epochs, n = 3							
Final fusion models, n = 3	X-axis force			X-axis vibration			Knife mark

**Table 5:** Performance comparison of the two network models on the test dataset.

	Indicators	Precision	Recall	F1
Status				
Improved residual dilated convolutional network				
Initial		0.9710	0.9699	0.9705
Normal		1.0000	0.9761	0.9879
Rapid		0.9696	0.9772	0.9734
Vibration		0.9825	0.9994	0.9909
Weighted average		0.9808	0.9806	0.9807
Multi-source data fusion network				
Initial		0.9983	0.9944	0.9964
Normal		1.0000	1.0000	1.0000
Rapid		0.9911	0.9972	0.9942
Vibration		0.9989	0.9967	0.9978
Weighted average		0.9971	0.9971	0.9971

**Table 6:** Performance comparison between fusion algorithms.

Fusion Method	Fusion Architecture	Fusion Data Sources	Accuracy	Average Inference Time
Uncertainty Weighting [33], 2018	Joint	7	96.46%	0.098 s
Dynamic Weight Averaging [34], 2019	Joint	7	95.15%	0.138 s
Dynamic Evidential Fusion [35], 2021	Collaborate	7	99.76%	0.176 s
Ours	Collaborate	7-4-3	99.20%	0.111 s
